# The EMIF-AD PreclinAD study: study design and baseline cohort overview

**DOI:** 10.1186/s13195-018-0406-7

**Published:** 2018-08-04

**Authors:** Elles Konijnenberg, Stephen F. Carter, Mara ten Kate, Anouk den Braber, Jori Tomassen, Chinenye Amadi, Linda Wesselman, Hoang-Ton Nguyen, Jacoba A. van de Kreeke, Maqsood Yaqub, Matteo Demuru, Sandra D. Mulder, Arjan Hillebrand, Femke H. Bouwman, Charlotte E. Teunissen, Erik H. Serné, Annette C. Moll, Frank D. Verbraak, Rainer Hinz, Neil Pendleton, Adriaan A. Lammertsma, Bart N. M. van Berckel, Frederik Barkhof, Dorret I. Boomsma, Philip Scheltens, Karl Herholz, Pieter Jelle Visser

**Affiliations:** 1 Alzheimer Center, Department of Neurology, VU University Medical Center, Neuroscience Amsterdam, PO Box 7057, 1007 MB Amsterdam, The Netherlands; 2 Wolfson Molecular Imaging Centre, Division of Neuroscience and Experimental Psychology, University of Manchester, Manchester, UK; 3 Department of Biological Psychology, VU University, Neuroscience Amsterdam, Amsterdam, The Netherlands; 4 Department of Ophthalmology, VU University Medical Center, Neuroscience Amsterdam, Amsterdam, The Netherlands; 5 Department of Radiology & Nuclear Medicine, VU University Medical Center, Neuroscience Amsterdam, Amsterdam, The Netherlands; 6 Neurochemistry Laboratory, Department of Clinical Chemistry, VU University Medical Center, Neuroscience Amsterdam, Amsterdam, The Netherlands; 7 Department of Clinical Neurophysiology, VU University Medical Center, Neuroscience Amsterdam, Amsterdam, The Netherlands; 8 Department of Internal Medicine, VU University Medical Center, Neuroscience Amsterdam, Amsterdam, The Netherlands; 9 Wolfson Molecular Imaging Centre, Division of Informatics, Imaging and Data Sciences, Faculty of Medicine, Biology and Health, University of Manchester, Manchester, UK; 10 Institutes of Neurology & Healthcare Engineering, UCL, London, UK; 11 Department of Psychiatry and Neuropsychology, School for Mental Health and Neuroscience, Alzheimer Center Limburg, Maastricht University, Maastricht, The Netherlands

**Keywords:** Preclinical Alzheimer’s disease, Amyloid, Cognitively normal, Monozygotic twins, [^18^F]flutemetamol

## Abstract

**Background:**

Amyloid pathology is the pathological hallmark in Alzheimer’s disease (AD) and can precede clinical dementia by decades. So far it remains unclear how amyloid pathology leads to cognitive impairment and dementia. To design AD prevention trials it is key to include cognitively normal subjects at high risk for amyloid pathology and to find predictors of cognitive decline in these subjects. These goals can be accomplished by targeting twins, with additional benefits to identify genetic and environmental pathways for amyloid pathology, other AD biomarkers, and cognitive decline.

**Methods:**

From December 2014 to October 2017 we enrolled cognitively normal participants aged 60 years and older from the ongoing Manchester and Newcastle Age and Cognitive Performance Research Cohort and the Netherlands Twins Register. In Manchester we included single individuals, and in Amsterdam monozygotic twin pairs. At baseline, participants completed neuropsychological tests and questionnaires, and underwent physical examination, blood sampling, ultrasound of the carotid arteries, structural and resting state functional brain magnetic resonance imaging, and dynamic amyloid positron emission tomography (PET) scanning with [^18^F]flutemetamol. In addition, the twin cohort underwent lumbar puncture for cerebrospinal fluid collection, buccal cell collection, magnetoencephalography, optical coherence tomography, and retinal imaging.

**Results:**

We included 285 participants, who were on average 74.8 ± 9.7 years old, 64% female. Fifty-eight participants (22%) had an abnormal amyloid PET scan.

**Conclusions:**

A rich baseline dataset of cognitively normal elderly individuals has been established to estimate risk factors and biomarkers for amyloid pathology and future cognitive decline.

**Electronic supplementary material:**

The online version of this article (10.1186/s13195-018-0406-7) contains supplementary material, which is available to authorized users.

## Background

Alzheimer’s disease (AD) is the most common cause of dementia and is characterized by amyloid plaques and neurofibrillary tangles with subsequently progressive neuronal loss and eventually death [1]. Aggregation of amyloid is supposed to be the first event in AD and starts years before cognitive impairment occurs [2–4]. Postmortem pathological and biomarker studies have demonstrated that 20–40% of cognitively normal elderly individuals possess abnormal amyloid levels in their brain [4–9]. These subjects are considered to be in the preclinical stage of AD [10, 11]. This presymptomatic window provides a unique opportunity for secondary prevention studies, as subjects have limited brain damage and no symptoms yet. Understanding the pathophysiological mechanisms underlying amyloid pathology in this preclinical stage of AD might also be critical to identify possible drug targets for the development of effective treatments.

There are, however, several research challenges for the development of prevention strategies in the preclinical AD stage. First, amyloid markers are needed for the diagnosis of preclinical AD. There is an urgent need for readily applicable screening markers, such as blood or imaging markers, to identify cognitively normal subjects at increased risk for amyloid pathology so that more expensive or invasive tests such as positron emission tomography (PET) scan or cerebrospinal fluid (CSF) via lumbar puncture can be performed in more selected populations. A number of markers have already been identified for this purpose but these need to be validated in preclinical/prodromal stages of the disease [12–15]. Second, there is still an incomplete understanding of what drives the development of amyloid pathology in cognitively normal subjects. Previous studies have identified a limited number of risk factors for amyloid pathology, such as Apolipoprotein E (APOE) genotype, age, and level of education [4, 16–18]. These established risk factors, however, can only explain part of the risk for amyloid pathology. Third, amyloid pathology has been associated with an increased risk for cognitive decline, but the rate of decline varies greatly [19]. A few possible prognostic factors in preclinical AD have been identified but they await replication [20, 21]. Fourth, current normative data for biomarkers and cognitive markers may be suboptimal as many cognitively normal subjects already have amyloid pathology. Finally, CSF and PET biomarkers for amyloid pathology do not match in about 15% of cases [22–24], in particular in cognitively normal subjects. It has been suggested that amyloid changes can be detected earlier in CSF than by PET but this requires further investigation [25].

In this paper, we describe the study design of the multisite PreclinAD study, which aims to address these clinical research challenges. Within this study, cognitively normal elderly individuals are recruited from the Manchester and Newcastle Age and Cognitive Performance Research Cohort (ACPRC) in Manchester [26] and the Netherlands Twin Register (NTR) in Amsterdam [27]. From the NTR we recruited monozygotic (MZ) twins. When a relation is observed between two markers, studying MZ twins enables exploring the nature of the observed relation: the MZ twin differences approach gives the possibility to study the relation excluding confounding by genetic factors (the twins are genetically identical); and the cross-twin cross-trait design, studying whether marker 1 in one twin can predict marker 2 in their co-twin, gives the opportunity to study the contribution of shared familial factors (genes and common environment) to the relation. Previous studies using AD-type dementia as an outcome estimated the amount of variance explained by genetic factors to be around 80% [28], suggesting a major genetic role in the development of AD. However, there is a lack of studies estimating the contribution of genetic and environmental influences on AD biomarkers in nondemented individuals and the role of environmental risk and protective factors for AD remains unclear [18].

**Fig. 1:**
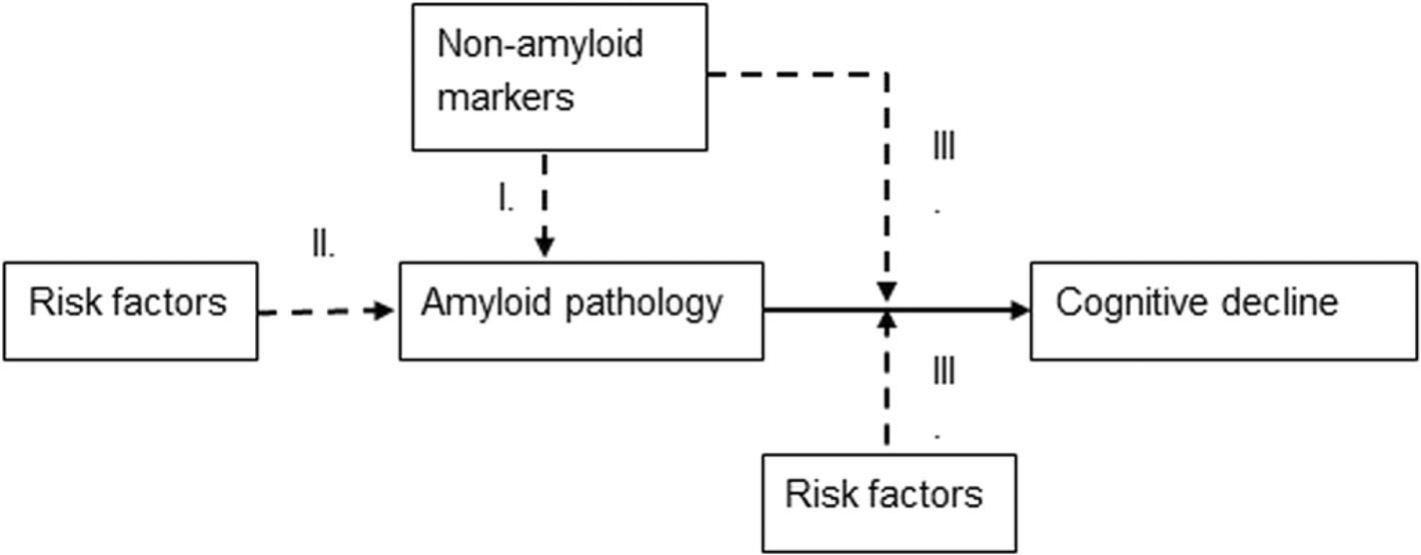
Hypothetical model of amyloid pathology. Hypothetical model for evaluating risk factors for amyloid pathology, for cognitive decline in subjects with amyloid pathology and other markers that might be involved in early AD pathology. (I) Markers for amyloid pathology in cognitively healthy elderly individuals; (II) risk factors for amyloid pathology; (III) prognostic markers for cognitive decline in cognitively normal subjects with amyloid pathology

The PreclinAD study aimed to: validate existing and discover new markers for amyloid pathology in cognitively normal elderly individuals; identify risk factors for amyloid pathology; identify prognostic markers for cognitive decline in cognitively normal subjects with amyloid pathology (Fig. 1); and determine the contribution of genetic and environmental influences on these markers.

## Methods

### Project

#### The European Medical Information Framework for AD

The study is part of the Innovative Medicine Initiative European Medical Information Framework for AD (EMIF-AD) project, which aims to facilitate the development of treatment for AD in nondemented subjects (http://www.emif.eu/) by discovering and validating diagnostic markers, prognostic markers, and risk factors for AD in nondemented subjects using existing data resources where possible.

### Sample selection

We included 81 cognitively normal participants from the ACPRC. The ACPRC originally comprised over 6000 adults from the North of England, UK, who underwent detailed batteries of cognitive function biannually until 2003 [26]. In 1999 and 2000, active members of this cohort were invited and consented to provide a deoxyribonucleic acid (DNA) sample to the Dyne-Steel DNA Archive for study of Cognitive Genetics in later life. In 2003, a subsample of 500 Manchester volunteers underwent detailed physical examination and provided samples of saliva, serum, and plasma. Over time, the cohort has reduced in size through attrition, largely by mortality, to a number of approximately 660 volunteers. Since 2003, study participants have been assessed biannually with a smaller battery of tests and rating scales in order to diagnose pathological cognitive impairment and emotional problems. The current study coincides with the fourth wave of follow-up investigations. In Amsterdam, monozygotic twins were recruited from the NTR [29]. The NTR started recruiting adolescent and adult twins and their relatives in 1987 and had included over 200,000 participants by 2012 [27]. Twins who gave consent for the NTR also allow researchers to approach them for participation in scientific studies. From 1991 onward, participants completed extensive questionnaires every 2 or 3 years and DNA was collected in the NTR-Biobank project [30]. Smaller subgroups of participants underwent biomarker collection such as laboratory tests, electroencephalogram, or magnetic resonance imaging (MRI) [31–33]. The current study is a new NTR sub study.

### Ethical considerations

The National Research Ethics Service Committee North West—Greater Manchester South performed ethical approval of the study in Manchester. The Medical Ethics Review Committee of the VU University Medical Center performed approval of the study in Amsterdam. Research was performed according to the principles of the Declaration of Helsinki and in accordance with the Medical Research Involving Human Subjects Act and codes on ‘good use’ of clinical data and biological samples as developed by the Dutch Federation of Medical Scientific Societies. All participants gave written informed consent.

### Inclusion criteria

Inclusion criteria for the PreclinAD cohort were age 60 years and older, a delayed recall score above − 1.5 SD of demographically adjusted normative data on the Consortium to Establish a Registry for Alzheimer’s Disease 10-word list [34, 35], a Telephone Interview for Cognitive Status-modified score of 23 or higher [36], a 15-item Geriatric Depression Scale score < 11 [37], and a Clinical Dementia Rating score of 0 [38] (Additional file 1: Table S1).

### Exclusion criteria

To avoid possible interference with normal cognition, subjects with the following medical conditions, at present or in the past, were excluded: diagnosis of mild cognitive impairment (MCI), probable AD or other neurodegenerative disorders such as Huntington disease, cortical basal degeneration, multiple system atrophy, Creutzfeldt-Jakob disease, primary progressive aphasia or Parkinson’s disease, stroke resulting in physical impairment, epilepsy with current use of antiepileptic drugs, brain infection (e.g., herpes simplex encephalitis), brain tumor, severe head trauma with loss of consciousness longer than 5 min, cancer with terminal life expectancy, untreated vitamin B12 deficiency, diabetes mellitus, thyroid disease, schizophrenia, bipolar disorders, or recurrent psychotic disorders. Furthermore, a history of recreational drug use, alcohol consumption > 35 units per week (1 unit = 10 ml or 8 g of pure alcohol), use of high-dose benzodiazepine, lithium carbonate, antipsychotics (including atypical agents), high-dose antidepressants, or Parkinson’s disease medication were exclusion criteria. Finally, subjects who were not able to attend the hospital due to physical morbidity or illness or who had a contraindication for MRI (e.g., metal implants, pacemaker, etc.) were excluded (Additional file 1: Table S1).

### Data collection

#### Neuropsychological testing battery and questionnaires

During a 4-h screening research facility visit (Manchester) or home visit (Amsterdam), participants underwent extensive neuropsychological testing and questionnaires. A complete overview of the neuropsychological testing battery and questionnaires is presented in Additional file 2: Table S2 and Additional file 3: Table S3, respectively. In short, we assessed memory function with the Rey auditory verbal learning task [39], visual association task [40], face–name associative memory examination [41], Rey complex figure recall [42], CANTAB paired associate learning [43], and digit span [44]. We also tested verbal fluency, naming [45], visuo-constructional skills, and executive functions [42, 46, 47] (see Additional file 2: Table S2). Using questionnaires we assessed social and physical activities [48–50], sleep quality [51, 52], activities of daily living [53, 54], memory complaints [55], and psychiatric symptoms [56] (see Additional file 3: Table S3).

#### Physical examination

Data on waist circumference, hip circumference, body mass index, resting blood pressure, heart rate, and grip strength of the dominant hand were collected for all participants (Table 1). In Manchester, an ankle/brachial pressure index and a 4-min walking test were also performed. In Amsterdam, a trained physician performed exploratory neurological examination. In addition, bioelectrical impedance analysis, repeated resting blood pressure measurement, and lead 1 of an electrocardiogram (measured by holding a Diagnostick [57] for 1 min) were performed and a color photograph of the face of each participant was taken. See Table 1 and Additional file 4: Table S4 for all biomarker data availability.

**Table 1:** Sample characteristics

Demographic	*n*	Combined sample	*n*	Amsterdam site	*n*	Manchester site
		(*n* = 285)		(*n* = 204)		(*n* = 81)
Age (years)	285	75.0 (9.7) (range 60–95)	204	70.8 (7.8) (range 60–94)	81	85.7 (4.3)*** (range 79–95)
Gender (% female)	285	182 (64%)	204	119 (58%)	81	63 (78%)**
Education (years)	278	14.8 (4.2)	204	14.9 (4.5)	74	14.2 (3.0)
NART	285	41.9 (6.0)	204	41.2 (6.4)	81	43.7 (4.3)***
MMSE	281	28.9 (1.2)	204	28.9 (1.2)	77	28.7 (1.3)
TICS-m	282	28.3 (3.2)	204	28.3 (3.0)	78	28.5 (3.7)
CERAD 10-word recall	285	22.8 (3.3)	204	22.0 (3.0)	81	24.8 (3.3)***
GDS	282	1.0 (1.5)	204	0.7 (1.2)	78	1.9 (1.7)***
CDR total	284	0 (0.1)	204	0	80	0.03 (0.1)*
CDR sum of boxes	284	0.03 (0.1)	204	0	80	0.1 (0.3)**
APOE e4 carrier	282	85 (30%)	202	66 (33%)	80	19 (24%)
APOE4 genotype	282		202		80	
e2e2		2 (1%)		2 (1%)		–
e2e3		24 (9%)		12 (6%)		12 (15%)
e2e4		9 (3%)		6 (3%)		3 (4%)
e3e3		171 (61%)		122 (60%)		49 (61%)
e3e4		69 (25%)		54 (27%)		15 (19%)
e4e4		7 (3)		6 (3%)		1 (1%)
Family history dementia	273	106 (39%)	203	92 (45%)	70	14 (20%)***
Diabetes type II	**–**	**–**	204	13 (6%)	*–*	*–*
Current smoker	281	23 (8%)	203	21 (10%)	78	2 (3%)
Alcohol use present	282	224 (79%)	204	158 (77%)	78	66 (85%)
Blood pressure (mmHg)	281	152 (21)/80 (12)	202	155 (21)/83 (11)	79	143 (19)/70 (10)***
Pulse rate (beats/min)	279	66 (11)	202	65 (11)	77	69 (10)**
Height (m)	283	1.66 (0.10)	204	1.69 (0.09)	79	1.60 (0.08)***
Weight (kg)	283	73.1 (14.0)	204	75.7 (13.6)	79	66.6 (13.0)***
Body mass index	283	26.3 (4.0)	204	26.4 (3.8)	79	26.1 (4.3)
Waist circumference (cm)	282	93.4 (13.6)	203	94.7 (12.0)	79	89.9 (16.6)**
Hip circumference (cm)	234	101.9 (11.4)	155	102.6 (9.8)	79	100.5 (14.0)
Grip strength (kg)	283	28.5 (11.3)	204	30.9 (10.9)	79	22.2 (9.8)***
CSF Aβ1–42 (pg/ml)	*–*	*–*	126	889 (314)	–	*–*
CSF Aβ1–40 (pg/ml)	*–*	*–*	126	9592 (2844)	–	*–*
Ratio CSF Aβ1–42/1–40	*–*	*–*	126	0.10 (0.03)	–	*–*
CSF total-tau (pg/ml)	–	–	126	412 (143)	–	*–*
CSF p-tau 181 (pg/ml)	–	–	126	76 (44)	–	*–*
Visual read PET abnormal	272	58 (22%)	196	32 (16%)	76	26 (34%)**
Fazekas score	279	1.3 (0.9)	199	1.2 (0.8)	80	1.7 (0.8)***
Medial temporal lobe atrophy score (average left and right)	277	0.7 (0.7)	197	0.6 (0.7)	80	0.9 (0.6)*
Parietal atrophy (average left and right)	279	1.1 (0.7)	199	1.1. (0.7)	80	1.2 (0.6)*

Data presented as mean (standard deviation) or *n* (%)

*NART* National Adult Reading Test, *MMSE* Mini-Mental State Examination, *TICS-m* Modified Telephone Interview for Cognitive Status, *CERAD* Consortium to Establish A Registry for Alzheimer’s Disease, *GDS* Geriatric Depression Scale, *CDR* Clinical Dementia Rating, *APOE* Apolipoprotein E, *CSF* cerebrospinal fluid, *Aβ* amyloid beta, *p-tau* phosphorylated tau, *PET* positron emission tomography

***p < 0.001, **p < 0.01, *p < 0.05, group difference assessed with *t* test or chi-square test

#### Blood collection

For all participants, 50 ml of blood was collected in the morning, after 2 h of fasting, including EDTA blood for DNA isolation, plasma, and buffy coat, clotted blood for serum, and Paxgene tubes for RNA isolation. Immediate plasma analysis was performed for complete blood count, hemoglobin A1C, 2-h fasting glucose, liver enzymes, lipid spectrum, C-reactive protein, erythrocyte sedimentation rate, thyroid stimulating hormone, and vitamin B12. EDTA tubes with anticoagulated whole blood were centrifuged at 1300–2000 × *g* for 10 min, and plasma and remaining buffy coat were, like whole blood for collecting serum, aliquoted according to the standardized operating procedures of the BIOMARKAPD project [58] in aliquots of 0.25–0.5 ml and stored locally until analysis. All samples were stored at − 80 **°**C within 2 h. Two 2.5-ml Paxgene tubes were stored at room temperature for a minimum of 2 h and a maximum of 72 h, before they were frozen at − 20 **°**C until RNA isolation. The EDTA whole blood tube for DNA analysis was stored at − 20 **°**C until isolation.

#### DNA and RNA collection

Extraction of DNA and RNA from peripheral blood samples was performed at both sites. In addition, at the Amsterdam site, buccal cells were collected for zygosity, genome-wide association studies, and epigenetics [59]. Amsterdam participants were genotyped on the Affymetrix Axiom array and the Affymetrix 6 array [60]; these were first cross-chip imputed following the protocols described by Fedko et al. [61] and then imputed into HRC with the Michigan Imputation server [62]. The APOE genotypes were assessed using isoforms in Manchester as described by Ghebranious et al. [63]. In Amsterdam, the APOE genotype was assessed using imputed dosages of the SNPs rs429358 (APOE ɛ4, imputation quality = 0.956) and rs7412 (APOE ɛ2, imputation quality = 0.729) [64].

#### Ultrasound carotid artery

In Manchester, a duplex ultrasound scan of the left and right carotid arteries was performed to collect data on velocity, vessel thickness, stenosis, and plaques rated according to the North American Symptomatic Carotid Endarterectomy Trial guidelines [65]. In Amsterdam, a duplex ultrasound scan of the right carotid artery was performed to assess intima media thickness and distension using ArtLab software [66–68].

#### Magnetic resonance imaging

##### Acquisition protocol

In Manchester, brain scans were performed at the Wellcome Trust Manchester Clinical Research Facility (Central Manchester University Hospital NHS Foundation Trust). All MRI investigations were performed on a 3 T Philips Achieva scanner using a 32-channel head coil. Participants underwent an MRI protocol that included 3D-T1, 3D fluid-attenuated inversion recovery (FLAIR), pseudocontinuous arterial spin labeling (ASL), and quantitative magnetization transfer scans. In Amsterdam, brain scans were also obtained using a 3 T Philips Achieva scanner equipped with an eight-channel head coil. The MRI protocol included structural 3D-T1, FLAIR, pseudocontinuous ASL, susceptibility weighted imaging (SWI), diffusion tensor imaging (DTI), and 6 min of resting state functional MRI (rs-fMRI). The MRI settings are presented in Additional file 5: Table S5.

##### Visual assessment

All MRI scans were reviewed for incidental findings by an experienced neuroradiologist, and visually rated by a single experienced rater (MtK) who was blinded to demographic information and twin pairing at the moment of rating. White matter hyperintensities were visually assessed on the FLAIR images using the 4-point Fazekas scale (none, punctuate, early confluent, confluent) [69]. Lacunes were defined as deep lesions from 3 to 15 mm with CSF-like signal on T1-weighted and FLAIR images. Microbleeds were assessed on SWI scans and defined as rounded hypointense homogeneous foci of up to 10 mm in the brain parenchyma. Medial temporal lobe atrophy was assessed on coronal reconstructions of the T1-weighted images using a 5-point visual rating scale [70]. Global cortical atrophy was rated on transversal FLAIR images using a 4-point scale [71]. Posterior cortical atrophy was assessed using a 4-point visual rating scale [72].

#### Amyloid positron emission tomography

##### [^18^F]flutemetamol

In both centers, [^18^F]flutemetamol was used as a fibrillar amyloid radiotracer. [^18^F]flutemetamol is an ^11^C-Pittsburgh compound B (PiB) derivative radiolabeled with ^18^F and has structural similarity to PiB, which is a frequently used compound for in-vivo detection of amyloid plaques [73]. In Manchester, the tracer [^18^F]flutemetamol was produced at the Wolfson Molecular Imaging Centre (WMIC) Good Manufacturing Practice radiochemistry facility using General Electric Healthcare’s (GEHC) FASTlab and cassettes. For Amsterdam, the same tracer was produced at the Cyclotron Research Center of the University of Liège (Liège, Belgium). GEHC was responsible for production and transportation of [^18^F]flutemetamol. Prior [^18^F]flutemetamol studies showed good brain uptake and radiation dosimetry similar to other radiopharmaceuticals in clinical use, test–retest variability for image quantitation differentiation between healthy participants and patients with AD, and the ability to detect brain Aβ [73].

##### Acquisition protocol

At both sites, all participants were scanned dynamically from 0 to 30 min and then again from 90 to 110 min after intravenous injection of 185 MBq (± 10%) [^18^F]flutemetamol. The initial scan (0–30 min) was shortened or omitted if it was not accepted or tolerated by the participant. The second time window (90–110 min) is the recommended interval for assessment of amyloid biomarker abnormality. In Manchester, all PET scans were performed on a high-resolution research tomograph brain scanner (HRRT; Siemens/CTI, Knoxville, TN, USA) at the WMIC of the University of Manchester. Two 7-min transmission scans using a ^137^Cs point source were acquired for subsequent attenuation and scatter correction; one prior to the first emission scan, and another following the second emission scan [74, 75]. In Amsterdam, all PET scans were performed using a Philips Ingenuity Time-of-Flight PET–MRI scanner at the Department of Radiology & Nuclear Medicine of the VU University Medical Center. Immediately prior to each part of the PET scan, a dedicated MR sequence (atMR) was performed for attenuation correction of the PET image [76]. For both sites, the first dynamic emission scan was reconstructed into 18 frames with progressive increase in frame length (6 × 5 s, 3 × 10 s, 4 × 60 s, 2 × 150 s, 2 × 300 s, 1 × 600 s). The second part of the scan consisted of 4 × 5-min frames. During scanning, the head was immobilized to reduce movement artifacts using laser beams.

##### Visual assessment

All [^18^F]flutemetamol amyloid PET scans were checked for movement and the frames were summed to obtain a static image (90–110 min). PET images were visually read as abnormal or normal by an experienced reader (SFC in Manchester and BNMvB in Amsterdam), blinded to clinical and demographic data, according to GEHC guidelines described in the summary of product characteristics [77].

#### CSF collection (Amsterdam site only)

Up to 20 ml of CSF was obtained by lumbar puncture in Sarstedt polypropylene syringes using a Spinocan 25-gauge needle in one of the intervertebral spaces between L3 and S1. One milliliter was immediately processed for leukocyte count, erythrocyte count, glucose, and total protein. The remaining CSF was mixed and centrifuged at 1300–2000 × *g* for 10 min at 4 **°**C. Supernatants were stored in aliquots of 0.25–0.5 ml and frozen within 2 h at − 80 **°**C and stored for future biomarker discovery studies [78]. Levels of amyloid β1–40 and β1–42 were analyzed using kits from ADx Neurosciences/Euroimmun according to the manufacturer’s instructions. All samples were measured in kits from the same lot.

#### Magnetoencephalography (Amsterdam site only)

Magnetoencephalography (MEG) measurements were recorded using a 306-channel, whole-head MEG system (ElektaNeuromag Oy, Helsinki, Finland) in a magnetically shielded room (Vacuumschmelze GmbH, Hanau, Germany). Participants were instructed to lie on a bed with their eyes closed but to stay awake and reduce eye movements in order to minimize artifacts. Participants were scanned for 5 min with eyes closed, 2 min with eyes open, and another 5 min with eyes closed. On MEG we used source-reconstructed time series (10.1016/j.neuroimage.2011.11.005) to extract both frequency spectrum properties (relative band power and peak frequency) and functional connectivity between regions, as well as network topology using modern network theory (synchronization likelihood, modularity, path length, phase lag index) [79, 80]. These analysis techniques were applied using BrainWave software (http://home.kpn.nl/stam7883/brainwave.html) [81] and inhouse MATLAB scripts (MATLAB Release 2012a; The MathWorks, Inc., Natick, MA, USA).

#### Ophthalmological markers (Amsterdam site only)

##### Exploratory eye examination

An exploratory eye examination including measurement of best corrected visual acuity, refractive error, and intra-ocular pressure (noncontact tonometry) was performed. In a subsample (*n* = 50), slit lamp examination by a trained physician was performed as well.

##### Ocular coherence tomography

Ocular coherence tomography (OCT) was performed using the Heidelberg Spectralis. With OCT we measured retinal nerve fiber layer tissue, total macular thickness, and the thickness of macular individual retinal layers using the built-in segmentation software from the Spectralis [82], which might correlate with cerebral amyloid pathology [83]. With the same device, fundus autofluorescence was performed to try to detect degenerative retinal abnormalities possibly related to amyloid pathology [83, 84].

##### Retinal imaging

Using a nonmydriatic camera (Topcon), two digital images (mostly 50°, and some 30°) per eye were taken of the retina—one centered to the macula, and the other to the optic nerve head—after pupil dilation with tropicamide. On the digitalized fundoscopy image we measured retinal vascular parameters using the Singapore I vessel Assessment software [85].

#### Data management

Data were stored in the online database CASTOR (https://castoredc.com/) with restricted access. Each site provided clinical information and sample information to the database according to a predefined case report form. Blood and CSF samples, PET and MRI scans, and MEG data are stored locally until centralized analysis.

### Follow-up visit

A follow-up visit including neuropsychological testing, questionnaires at both sites, and physical examination, blood sampling, buccal cell collection, and lumbar puncture in a subset will be performed after 21 months ± 3 months.

Follow-up started in February 2017 and is still ongoing. So far 241 individuals have been invited, and of those 221 (92%) participated in the follow-up.

For the twin pairs, an additional follow-up visit after 4 years is planned, starting in January 2019. This follow-up includes amyloid-PET, tau-PET, MRI, lumbar puncture, neuropsychological testing, questionnaires, physical examination, blood sampling, and buccal cell collection.

### Statistical approaches

#### Group analysis

The main outcome measure will be the presence of amyloid pathology as a dichotomous and continuous outcome measure. We aim to identify for each diagnostic modality the best set of predictors for amyloid pathology using step forward selection. The best predictors for each modality will be combined in a single risk score, based on the β value of these predictors in the regression model. Analysis will be performed using multivariate multilevel generalized estimating equation analysis with correction for age, gender, education, and twin status (Amsterdam only) [86]. In addition, as there are differences between the cohorts, we will correct for cohort in the analysis and test interactions of predictor variables with cohort to check whether pooling the data may introduce a bias.

## Results

### Inclusion

#### Manchester

**Fig. 2:**
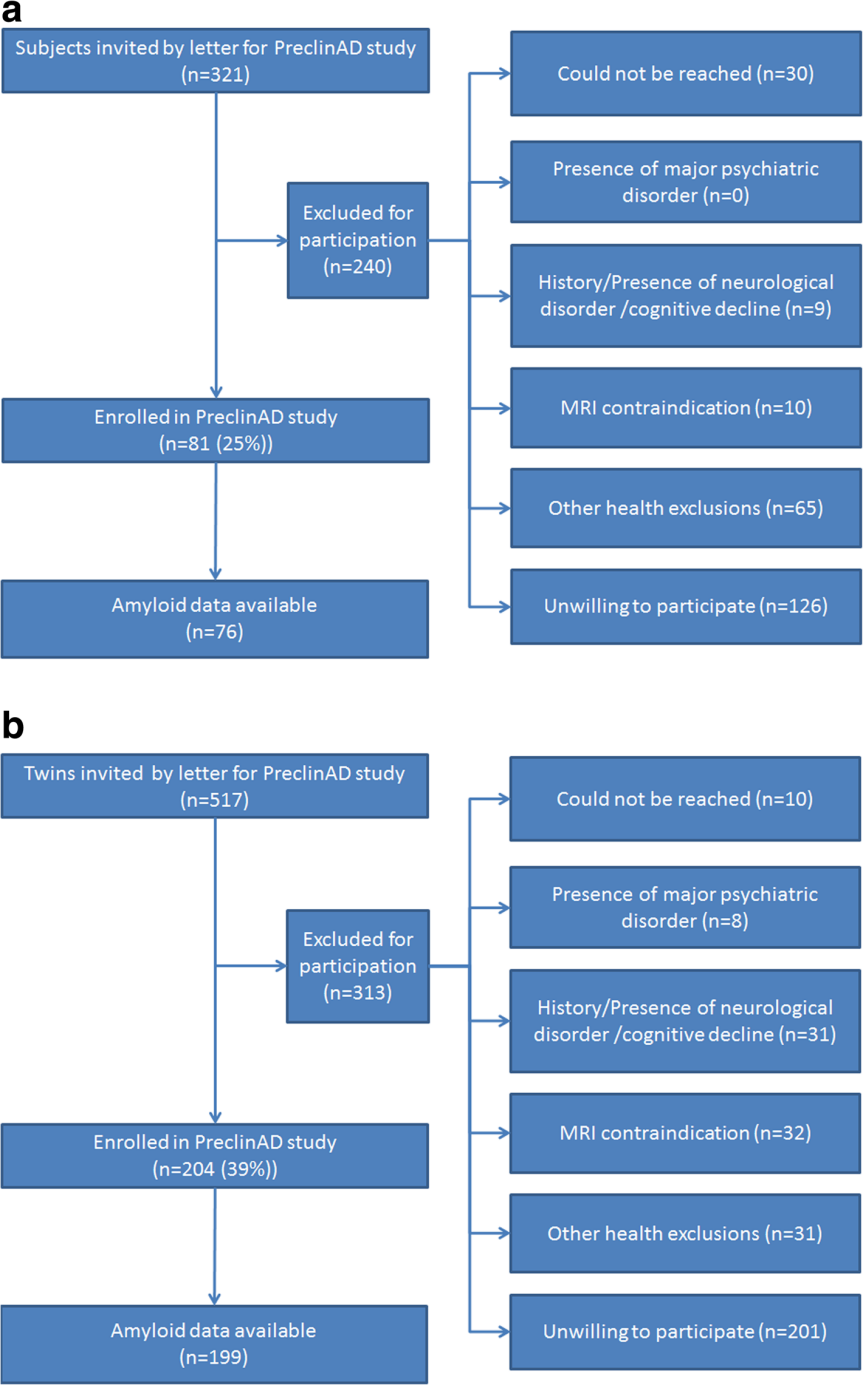
Inclusion flow chart for participants from **a** Manchester invited subjects selected from a sample of 660 subjects who were part of Manchester and Newcastle Age and Cognitive Performance Research Cohort (ACPRC, Manchester) at time of recruitment and **b** from Amsterdam invited twins selected from a sample of 678 monozygotic twins who were actively registered in Netherlands Twin Register (Amsterdam) at time of recruitment

From the ACPRC, 321 subjects in total were invited by letter to participate in the PreclinAD study. From this selection, 81 subjects were included for participation (see Fig. 2a).

#### Amsterdam

In total 517 twins from the NTR were invited by letter. Of these, 100 complete pairs (99 MZ, one dizygotic, as confirmed with DNA analysis) and four singletons, of which the co-twin did not meet the inclusion criteria due to cognitive impairment of other neurological conditions, were included (see Fig. 2b). This also included one twin who appeared to be demented at baseline hospital visit, even though this subject passed the inclusion criteria at first, and one subject from a monozygotic triplet, which we included due to the unique opportunity to analyze a genetically identical triplet, but this subject did not meet the inclusion criteria due to MCI. All participants, except for one twin pair, have European descent. When analyzing genetic data this twin pair will be excluded from the analysis.

### Demographics and biomarkers

Participants were on average 74.8 years old, 64% female, and 30% APOE ε4 carriers; for further baseline characteristics see Table 1. Participants tested in Manchester were older compared to Amsterdam participants (85.7 vs 70.8 years, *p* < 0.001) and more often female (78 vs 58%, *p* < 0.01). Manchester participants also had a higher intelligence score according to the Adult Reading Task (43.7 vs 41.2, *p* < 0.001), less often a family member with dementia (20 vs 45%, *p* < 0.001), lower blood pressure, (143/70 vs 155/83 mmHg, *p* < 0.001), and higher white matter lesion load according to the Fazekas score (1.7 vs 1.2, *p* < 0.001) (Table 1).

Amyloid data were available for 275 participants (Manchester *n* = 76, Amsterdam *n* = 199). In Amsterdam, 123 participants had both CSF and PET available, 73 PET only, and three CSF only. For 10 participants we were unable to assess their amyloid status: six participants were not able to attend the hospital after inclusion, one participant did not undergo PET due to meningiomas on MRI, two participants suffered from claustrophobia during the hospital visit, and one participant had a panic attack before injection of the PET tracer. Dynamic PET scans were present in 261 participants: four participants failed their dynamic scan due to logistic problems, and in seven participants quality control of the images failed.

### Amyloid pathology

**Fig. 3:**
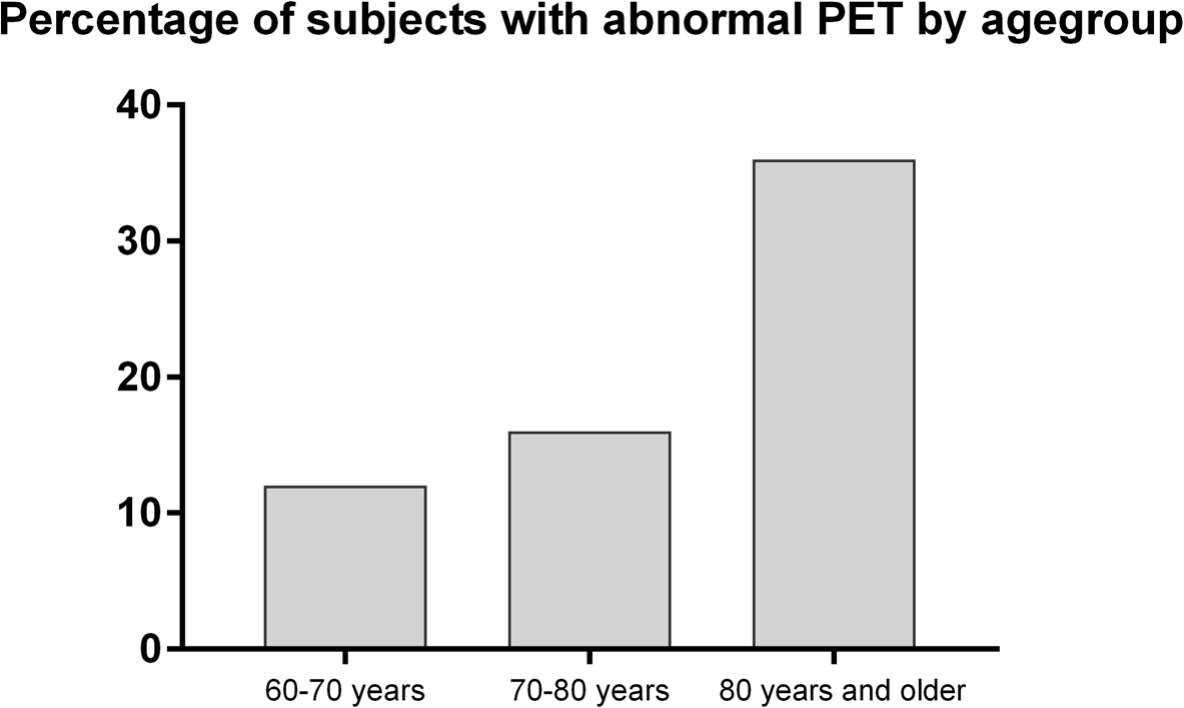
Amyloid abnormality on PET scan per age group (*n* = 58, 22%). Abnormal PET scan visually read on summed static PET images: 12% of subjects aged 60–70 years, 16% of subjects between 70 and 80 years, and 36% of subjects 80 years and older had abnormal PET scan

Of the 272 participants with a static PET amyloid measure available, 58 (21%) had an abnormal PET scan as visually read on a summed static PET image. An abnormal PET was less common in Amsterdam (16%) than in Manchester (34%) (*p* < 0.001). The prevalence of abnormal amyloid PET scans was higher in older age groups (Fig. 3).

## Discussion

The PreclinAD study is a prospective cohort study of 285 cognitively normal elderly individuals with extensive phenotyping for amyloid pathology, neurodegeneration markers, cognition, and lifestyle factors.

We noted some differences in baseline characteristics between the Manchester and Amsterdam sites. This could mainly be explained by the higher age in the Manchester substudy. The prevalence of amyloid pathology increased with age, although the prevalence was somewhat lower than would be expected based on a large subject-level meta-analysis, in particular in the age range below 80 years [4]. This might be explained by the relatively healthy sample of participants, due to the strict inclusion and exclusion criteria.

The Amsterdam sub study is the first to assess a wide range of AD markers in a large sample of cognitively normal monozygotic twin pairs above age 60 years. The uniqueness of studying a cohort of twin pairs sharing 100% of their genetic material enables us to further explore the nature of the relation between AD markers. If MZ twin pairs are highly similar for AD markers, this suggests involvement of shared genetic and/or shared environmental factors, whereas within-pair differences indicate the involvement of unique environmental factors [87]. The strength of the MZ twin within-pair difference model further enables us to identify environmental risk factors (e.g., smoking, alcohol use, diet, sleep, physical activity, cognitive activity, and education) that, either directly or indirectly through epigenetic mechanisms, explain observed differences in AD markers within pairs. This may provide clues for novel preventive and therapeutic strategies. However, this model also has the disadvantage that, because MZ twins are genetically identical, we have to correct for twin dependency in all analysis, which may reduce statistical power [86]. Further, we did not include dizygotic twins in the current study, because this optimizes power for twin difference analysis, thereby strengthening the search for environmental risk factors influencing AD development. However, this has the disadvantage that the relative contribution of shared genetic and shared environmental factors to within-pair correlations cannot be estimated. Still, previous studies in elderly twins suggested that the contribution of shared environment at older age is highly limited, possibly because subjects have already been living apart for a longer period of time [88, 89].

A strength of our study, compared to other studies on preclinical AD, is that participants have been recruited from cohorts that have been ongoing for up to 20 years, which provides the possibility to test biomarkers, cognition, and lifestyle collected in the past as predictors for AD biomarkers. Our study design also has several limitations. First, although acquisition protocols were harmonized across sites, they were not always identical (e.g., use of HRRT vs PET-MR). For this reason, site will be used as a covariate in all analyses. Some of the biomarkers were only acquired at the Amsterdam site, which will reduce the statistical power for the analysis of these markers.

## Conclusions

We collected a large European cognitively normal sample with an extensive panel of AD biomarkers available at baseline, with clinical follow-up planned after 2 years, to identify healthy elderly individuals at risk for amyloid pathology and future cognitive decline. Results from this study will improve understanding of the pathophysiology of AD and thereby help to adapt the design of secondary prevention trials.

## Additional files

Additional file 1: **Table S1.** Inclusion and exclusion criteria. (DOCX 36 kb)

Additional file 2: **Table S2.** Neuropsychological tests baseline. (DOCX 48 kb)

Additional file 3: **Table S3.** Questionnaires baseline. (DOCX 37 kb)

Additional file 4: **Table S4.** Biomarkers baseline. (DOCX 24 kb)

Additional file 5: **Table S5.** MRI settings. (DOCX 15 kb)

## Abbreviations

ACPRC: Manchester and Newcastle Age and Cognitive Performance Research Cohort
AD: Alzheimer’s disease
APOE: Apolipoprotein E
ASL: Arterial spin labeling
CSF: Cerebrospinal fluid
DTI: Diffusion tensor imaging
EDTA: Ethylene diamine tetra-acetic acid
EMIF-AD: European Medical Information Framework for AD
FLAIR: 3D fluid-attenuated inversion recovery
GEHC: General Electric Healthcare
HRRT: High-resolution research tomograph
MCI: Mild cognitive impairment
MEG: Magnetoencephalography
MRI: Magnetic resonance imaging
MZ: Monozygotic
NTR: Netherlands Twins Register
OCT: Ocular coherence tomography
PET: Positron emission tomography
rs-fMRI: Resting state functional MRI
SWI: Susceptibility weighted imaging
WMIC: Wolfson Molecular Imaging Centre
