# Supplementary material for: The EMIF-AD PreclinAD study: study design and baseline cohort overview
Source: Alzheimers Res Ther. 2018 Aug 4;10:75. doi: 10.1186/s13195-018-0406-7 (PMC6091034; doi:10.1186/s13195-018-0406-7)
Supplement: Supplementary file 1 — Table S1. Inclusion and exclusion criteria. (DOCX 36 kb) [file 13195_2018_406_MOESM1_ESM.docx]

**Additional table 1 Inclusion and exclusion criteria**

| *Inclusion criteria*  – Age ≥60 years  – TICS-m[36] >22  – CERAD 10 word list immediate and delayed recall[39] > -1.5 SD of age adjusted normative data  – GDS-15[37] <11  – CDR[38] 0 | |
| --- | --- |
| *Exclusion criteria*  – Clinical diagnosis of MCI or probable AD at baseline  – Severe head trauma with loss of consciousness  – Brain tumor (past, present)  – Schizophrenia, bipolar disorders or recurrent psychotic  disorders  – Stroke resulting in cognitive impairment  – Neurodegenerative diseases such as Parkinson’s disease and Huntington’s disease  – Epilepsy, currently using anti-epileptic drugs  – Brain infections (acute or a sequel of infection)  – Cancer with terminal life expectancy  – Known vitamin B12 deficiency without treatment  – Uncontrolled diabetes mellitus | – Known thyroid disease without treatment  – History of recreational drug use  – Alcohol consumption >35 units per week  – Physical morbidity or illness which will not permit attendance at visit sessions  – Contraindication for MRI (e.g. metal implants, pacemaker etc.)  – Medications that may impair cognition, at the discretion of the investigator, e.g.:   - High dose benzodiazepine - Lithium carbonate - Antipsychotics including atypical agents - High dose antidepressants - Parkinson’s disease medicines |

*TICS-m: Modified Telephone Interview for Cognitive Status; CERAD: Consortium to Establish A Registry for Alzheimer's Disease; GDS-15: 15-item Geriatric Depression Scale; CDR: Clinical Dementia Rating Scale; MCI: Mild cognitive impairment; AD: Alzheimer’s disease; MRI: Magnetic resonance imaging*
