# Supplementary material for: The EMIF-AD PreclinAD study: study design and baseline cohort overview
Source: Alzheimers Res Ther. 2018 Aug 4;10:75. doi: 10.1186/s13195-018-0406-7 (PMC6091034; doi:10.1186/s13195-018-0406-7)
Supplement: Supplementary file 2 — Table S2. Neuropsychological tests baseline. (DOCX 48 kb) [file 13195_2018_406_MOESM2_ESM.docx]

**Additional table 2** **Neuropsychological tests baseline**

| Neuropsychological Test | Range | Participants completed | Mean (SD) Amsterdam | Participants completed | Mean (SD) Manchester |
| --- | --- | --- | --- | --- | --- |
| RAVLT |  |  |  |  |  |
| -       Immediate recall | 0-75 | 197 | 42 (9.2) | 77 | 49 (8.9) |
| -       Delayed recall 20 minutes | 0-15 | 196 | 8.4 (2.9) | 77 | 10.7 (3.3) |
| -       Learning | 0-15 | 197 | 6.1 (2.0) | 77 | 7.6 (2.1) |
| - Recognition | 0-30 | 196 | 28.3 (2.1) | NA | - |
| WAIS-III digit span |  | 204 |  | 77 |  |
| - Forward Score | 0-14 |  | 8.6 (1.9) |  | 10.2 (2.2) |
| - Forward Score | 0-8 |  | 5.8 (1.1) |  | 6.7 (1.3) |
| - Backwards Score | 0-14 |  | 5.9 (1.6) |  | 6.9 (1.9) |
| - Backwards Span | 0-8 |  | 4.4 (1.0) |  | 4.9 (1.1) |
| FNAME |  | 182 |  | 79 |  |
| - Total names | 0-48 |  | 19.6 (10.1) |  | 18.8 (11.2) |
| - Total occupation | 0-48 |  | 32.8 (9.0) |  | 30.0 (10.0) |
| - Total names and occupation | 0-96 |  | 52.4 (17.6) |  | 49.4 (20.2) |
| Rey Complex Figure Test |  |  |  |  |  |
| -       Copy | 0-36 | 204 | 33.5 (3.4) | 77 | 34.3 (3.0) |
| -       Delayed copy 3min | 0-36 | 204 | 18.3 (5.5) | NA | - |
| -       Delayed copy 20min | 0-36 | 202 | 18.2 (5.4) | 77 | 14.9 (6.2) |
| Verbal Fluency |  |  |  |  |  |
| - In English testing the letters F, A, and S, in Dutch the letters D, A and T |  | 204 | 37.6 (10.4) | 77 | 49.6 (12.3) |
| - Category fluency animal 1 minute |  | 204 | 22.0 (7.1) | 76 | 18.8 (4.6) |
| - Category fluency animal 2 minutes |  | 204 | 34.6 (9.3) | NA | - |
| - Category fluency fruits |  | NA | - | 77 | 13.5 (3.9) |
| - Category fluency birds |  | NA | - | 77 | 14.4 (4.7) |
| - Category fluency household items |  | NA | - | 77 | 19.9 (4.9) |
| - Category fluency tools |  | NA | - | 77 | 10.4 (3.2) |
| - Category fluency vehicles |  | NA | - | 77 | 12.0 (3.1) |
| TMT A & B |  |  |  |  |  |
| - TMT A | seconds | 204 | 43.3 (21.3) | 77 | 42.4 (16.0) |
| - TMT A errors |  | 204 | 0.2 (0.4) | NA | - |
| - TMT B | seconds | 203 | 107.3 (63.7) | 77 | 88.9 (36.0) |
| - TMT B errors |  | 203 | 0.6 (1.1) | NA | - |
| Graded naming | 0-30 | 203 | 18.2 (3.7) | 77 | 24.7 (3.5) |
| CANTAB |  |  |  |  |  |
| - RVP-A |  | 178 | 8411 (1742) | 72 | 8585 (638) |
| - RVP Median response latency |  | 178 | 912 (1253) | 72 | 560 (142) |
| - PAL Total errors adjusted |  | 203 | 28.8 (16.3) | 75 | 33.9 (17.7) |
| - RTI Simple median RT |  | 203 | 299 (61) | 75 | 310 (50) |
| - RTI Five choices median RT |  | 203 | 333 (48) | 75 | 358 (47) |
| - RTI SD five choices RT |  | 203 | 61 (36) | 75 | 67 (23) |
| - SWM Between errors |  | 200 | 21 (9.4) | 74 | 22 (7.8) |
| - SWM Strategy |  | 200 | 18 (2.5) | 74 | 18 (2.8) |
| WAIS-R DSST | 0-93 | 201 | 45.1 (12.1) | NA | - |
| Savage alphabet coding task (LLS) |  | NA |  | 81 |  |
| - Trial 1 Correct |  |  | - |  | 42 (11) |
| - Trial 2 Correct |  |  | - |  | 46 (11) |
| - Trial 3 Correct |  |  | - |  | 48 (11) |
| - Trial 4 Correct |  |  | - |  | 49 (11) |
| - 2 minute recall |  |  | - |  | 4 (3) |
| VAT |  | 204 |  | NA |  |
| -       A 2 trials | 0-12 |  | 12 (0.8) |  | **-** |
| -       B 2 trials | 0-12 |  | 11 (1.3) |  | **-** |
| -       Naming | 0-12 |  | 12 (0.2) |  | **-** |
| One minute reading task | 1-145 | 204 | 89 (16) | NA | **-** |
| Klepel B | 1-145 | 204 | 82 (21) | NA | **-** |
| Heim intelligence test (AH4) |  | NA |  | 81 |  |
| - Number attempted part 1 |  |  | - |  | 40 (10) |
| - Answers correct part 1 |  |  | - |  | 33 (10) |
| - Number attempted part 2 |  |  | - |  | 40 (10) |
| - Answers correct part 2 |  |  | - |  | 32 (9) |
| Memory circle test |  | NA |  | 81 |  |
| - Correct name, correct segment | 0-24 |  | - |  | 3.8 (2.1) |
| - Correct name, wrong segment | 0-12 |  | - |  | 5.5 (1.6) |
| WAIS vocabulary test | 0-74 | NA | - | 81 | 58 (11) |
| ACE TOTAL | 100 | NA | - | **77** | 92 (5) |

*RAVLT: Rey auditory verbal learning test, TMT: Trail Making Test, VAT: Visual Association Test, WAIS: Wechsler Adult Intelligence Scale, DSST: Digit Symbol Substitution Task, CANTAB: Cambridge Neuropsychological Test Automated Battery, RVP: Rapid Visual information Processing, PAL: Paired Associate Learning, RTI: Reaction Time, SWM: Spatial-working Memory, F-NAME: Face-name associative memory exam*
