# Supplementary material for: The EMIF-AD PreclinAD study: study design and baseline cohort overview
Source: Alzheimers Res Ther. 2018 Aug 4;10:75. doi: 10.1186/s13195-018-0406-7 (PMC6091034; doi:10.1186/s13195-018-0406-7)
Supplement: Supplementary file 3 — Table S3. Questionnaires baseline. (DOCX 37 kb) [file 13195_2018_406_MOESM3_ESM.docx]

**Additional table 3** Q**uestionnaires baseline**

| Questionnaires | Range | Participants  completed | Mean (SD)  Amsterdam | Participants  completed | Mean (SD)  Manchester |
| --- | --- | --- | --- | --- | --- |
| CDR - memory | 0-3 | 204 | 0 | 80 | 0.03 (0.11) |
| PASE (self-reported) | 7-60 | 204 | 27.9 (5.3) | 77 | 23.8 (5.1) |
| CCI (self-reported) | 20-100 | 204 | 23 (3.5) | 74 | 36.1 (13.1) |
| CCI (informant-reported) | 20-100 | 204 | 22 (3.7) | 62 | 31.6 (9.6) |
| FAQ (informant-reported) | 0-30 | 204 | 0.2 (0.7) | 76 | 1.2 (2.6) |
| AD8 (informant-reported) | 0-8 | 203 | 0 (0.2) | 77 | 0.6 (1.0) |
| Berlin Sleep questionnaire  (self-reported) | high/low | 204 | 49/155 | NA | - |
| Cognitive abilities questionnaire  (self-reported) Manchester version | 1-48 | NA | - | 65 | 21 (3.9) |
| Cognitive abilities questionnaire  (self-reported) Amsterdam version | 26-164 | 204 | 90 (17.4) | NA | - |
| NPI-q (informant-reported) | 0-96 | 204 | 1 (3.1) | NA | - |
| Amsterdam iADL (informant-reported, t-score) | 0-100 | 199 | 69.4 (4.4) | NA | - |

*CDR: Clinical Dementia Rating Scale; PASE: Physical Activity Scale for the Elderly; CCI: Cognitive Complaints Index; FAQ: Functional Activities Questionnaire; AD8: Ascertain Dementia 8; NPI-q: Neuropsychiatric Inventory Questionnaire; iADL: instrumental Activities of Daily Living*
