# Supplementary material for: The EMIF-AD PreclinAD study: study design and baseline cohort overview
Source: Alzheimers Res Ther. 2018 Aug 4;10:75. doi: 10.1186/s13195-018-0406-7 (PMC6091034; doi:10.1186/s13195-018-0406-7)
Supplement: Supplementary file 4 — Table S4. Biomarkers baseline. (DOCX 24 kb) [file 13195_2018_406_MOESM4_ESM.docx]

**Additional table 4** Biomarkers baseline

| Biomarker |  | Amsterdam participants completed | Manchester participants completed |  |  |  |
| --- | --- | --- | --- | --- | --- | --- |
| Physical measures | | | |  |  |  |
| - Bioelectrical Impedance Analysis [86] | Muscle, fat and weight analysis | 144 | NA |  |  |  |
| - Neurological examination |  | 197 | NA |  |  |  |
| - Lead 1 electrocardiogram | Assessed with Diagnostick [57] | 169 | NA |  |  |  |
| - Color photograph face |  | 197 | NA |  |  |  |
| - Ophthalmological examination | Slit lamp  Eye pressure  Refraction | 50  131  123 | NA |  |  |  |
| Fluid specimens | | | |  |  |  |
| - Blood | 50 mL clotted blood and plasma | 204 | 76 |  |  |  |
| - Cerebrospinal Fluid | maximum of 20 mL | 126 | NA |  |  |  |
| - Buccal cells |  | 194 | NA |  |  |  |
| Imaging | | | |  |  |  |
| - [^18^F]flutemetamol PET imaging |  | PET-MRI scanner | HRRT scanner |  |  |  |
| - Dynamic 0-30 minutes |  | 195 | 74 |  |  |  |
| - Dynamic 90-110 minutes |  | 197 | 76 |  |  |  |
| - Magnetic Resonance Imaging |  | 3T Philips Achieva | 3T Philips Achieva |  |  |  |
|  |  | 8 coil | 32 coil |  |  |  |
| - 3DT1 |  | 198 | 81 |  |  |  |
| - 3D Fluid-attenuated inversion recovery |  | 199 | 81 |  |  |  |
| - pseudo continuous Arterial Spin Labeling |  | 197 | 80 |  |  |  |
| - Susceptibilty Weighted Imaging |  | 198 | NA |  |  |  |
| - Diffusion Tensor Imaging |  | 197 | NA |  |  |  |
| - Resting state functional MRI |  | 191 | NA |  |  |  |
| - quantitative Magnetization Transfer |  | NA | 80 |  |  |  |
| - Ultrasound carotid arteries |  | right | bilateral |  |  |  |
| - Intima Media Thickness |  | 155 | 60 |  |  |  |
| - Distension |  | 127 | NA |  |  |  |
| - Stiffness |  | NA | 64 |  |  |  |
| - Velocity |  | NA | 65 |  |  |  |
| - Stenosis present? |  | NA | 64 |  |  |  |
| - Magneto Encephalography |  | 187 | NA |  |  |  |
| - Ocular Coherence Tomography |  | 187 | NA |  |  |  |
| - Fundus image |  | 165 | NA |  |  |  |
|  |  |  |  | |  |  |
