# Supplementary material for: The EMIF-AD PreclinAD study: study design and baseline cohort overview
Source: Alzheimers Res Ther. 2018 Aug 4;10:75. doi: 10.1186/s13195-018-0406-7 (PMC6091034; doi:10.1186/s13195-018-0406-7)
Supplement: Supplementary file 5 — Table S5. MRI settings. (DOCX 15 kb) [file 13195_2018_406_MOESM5_ESM.docx]

**Additional table 5** MRI settings

| MRI sequence | Settings |
| --- | --- |
| 3DT1 | Sagittal turbo field echo sequence (1.00 mm x 1.00 mm x 1.00 mm voxels, TR = 7.9 ms, TE = 4.5 ms, FA = 8 degrees) |
| 3D Fluid-attenuated inversion recovery | 3D sagittal fat-saturated sequence (1.12 mm x 1.12 mm x 1.12 mm voxels, TR = 4800 ms, TE = 279 ms, inversion time = 1650 ms) |
| pseudo continuous Arterial Spin Labeling | Perfusion images (3.0 mm x 3.0 mm x 6.0 mm voxels, labeling time = 1650 ms, postlabel delay = 2025 ms , TR = 4560 ms, TE = 14 ms, acquisition time = 4 minutes) |
| Susceptibilty Weighted Imaging | A 3D transversal scan (0.8 mm x 0.8 mm x 1.20 mm voxels, TR = 19 ms, TE=27 ms, FA = 8 degrees) |
| Diffusion Tensor Imaging | Spin EPI scan (32 directions, b value = 1000, 2.00 mm x 2.00 mm x 2.00 mm voxels, TR = 7517 ms, TE = 92 ms, FA = 90 degrees) |
| Resting state functional MRI | Fast field echo EPI sequence (3.30 mm x 3.30 mm x 3.00 mm voxels, TR = 1800 ms, TE = 35 ms, FA = 80 degrees) |
| quantitative Magnetization Transfer | 3D fast field echo acquisition with a 112x112x60 matrix (2.0 mm x 2.0 mm x 2.0 mm voxels, TR = 38.15ms TE = 1.39 ms), 8 acquisitions of differing offset frequency and flip angle |

*TR: repetition time; TE: echo time; FA: flip angle; EPI: echo planar imaging*
